# Direction control of quasi-stokeslet induced by thermoplasmonic heating of a water vapor microbubble

**DOI:** 10.1038/s41598-019-41255-5

**Published:** 2019-03-18

**Authors:** Kyoko Namura, Souki Imafuku, Samir Kumar, Kaoru Nakajima, Masaaki Sakakura, Motofumi Suzuki

**Affiliations:** 1 Department of Micro Engineering, Kyoto University, Kyoto Daigaku-Katsura, Nishikyo-ku, Kyoto 615-8540 Japan; 2 University of Southampton, Optoelectronics Research Centre, Southampton, SO17 1BJ United Kingdom

## Abstract

We investigate the control of flow direction around a water vapor bubble using the thermoplasmonic effect of a gold nanoisland film (GNF) under laser irradiation with multiple spots. By focusing a laser spot on the GNF immersed in degassed water, a water vapor bubble with a diameter of ~10 μm is generated. Simultaneously, a sub laser spot was focused next to the bubble to yield a temperature gradient in the direction parallel to the GNF surface. Consequently, rapid flow was generated around the bubble, whose flow direction was dependent on the power of the sub laser spot. The observed flow was well-described using a stokeslet; the latter contained components normal and parallel to the GNF surface and was set to 10 μm above the GNF. This technique allows us to apply a significant force on the microfluid at the vicinity of the wall in the direction parallel to the wall surface, where the flow speed is generally suppressed by viscosity. It is expected to be useful for microfluidic pumping and microfluidic thermal management.

## Introduction

During the last several decades, microfluidics has demonstrated significant impact on biological and chemical applications through the development of lab-on-a-chip devices^1,2^ and micro total analysis systems^3,4^. Because these devices treat fluids with a volume of the order of a microliter or even smaller, they have helped significantly reduce the analytical time and cost compared to those required in conventional bench-top methods. Microfluidics have also attracted much attention in the field of thermal engineering to fulfill the urgent need of cooling systems for electronic devices^5,6^. Thin microfluidic channels are expected to be integrated with electrical circuits to improve their heat dissipation. Although the small volume of the working fluid yields these significant benefits, it is not trivial to pump the fluid packed in thin fluidic channels^7–9^. This is primarily because of the large pressure drop^10^ and difficulty in applying a force to the liquids packed in the long and thin fluidic channels, where flow is dominated by viscosity. One of the potential microfluidic pumping techniques is thermally controlled bubble-based pumping. Gas bubbles can be generated by heating the liquid and removed by cooling it owing to the phase transition and diffusion of dissolved gasses in water. These processes involve large volume changes in the bubbles and induce liquid flow around them^11–13^. In addition, the temperature gradient on the bubble surface may induce surface tension differential, and subsequently generate flow around the bubble, namely the Marangoni (or thermocapillary) flow^14,15^. This flow becomes prominent compared to the flow induced by volume forces such as gravity at the micrometer scale, where the surface-to-volume ratio becomes large^16–22^. Therefore, in microfluidic channels, the bubbles resemble thermally controlled mechanical components that can be created and removed. They are expected to be useful for the realization of flexible and powerful microfluidic pumping tools. However, the precise control of the flow around the microbubble has been difficult using the position-fixed heaters, such as a wire heater. This is because the preparation of microbubbles with the desired position and size at the desired growth rate is difficult. In addition, such uncertainties of microbubble position and size render it difficult to control the Marangoni flow, which is sensitive to the temperature gradient along the bubble surface.

For the realization of the spatio-temporally flexible bubble generation and its temperature control, the thermoplasmonic effect of noble metal nanoparticles has attracted interest recently^23–30^. Noble metal nanoparticles absorb light energy and convert it to heat energy within several picoseconds^31,32^. Therefore, a laser spot on a noble metal nanoparticle film can be used as a mobile and localized heat source. Recently, we have reported a rapid flow generation around a water vapor microbubble in degassed water using the thermoplasmonic effect of a gold nanoisland film (GNF)^33^. By focusing a laser on the GNF immersed in degassed water, a water vapor microbubble with a diameter of ~10 μm was generated on the laser spot. Under continuous heating, the water vapor bubble involved significantly rapid flows compared to an air bubble generated in non-degassed water. In addition, the flow field around the water vapor bubble was well described using a point force, i.e., a stokeslet that was set normal to the GNF surface, from which the flow speed was estimated to exceed 1 m/s in the vicinity of the bubble. This flow speed is extremely large as compared to that can be typically achieved in conventional microfludic channels by using syringe pumps, which is of the order of 0.1–10 mm/s^34–36^. This technique is attractive for realization of ubiquitous microfluidic mixers and pumps because such an rapid flow can be generated at an arbitrary spot of the microchannels by focusing laser on the GNF. However, the present technique is not best suited for pumping because flow generated around the water vapor bubble is axisymmetric around the normal to the substrate surface. To pump microfluids in a thin channel, it is better to generate flows in the direction parallel to the substrate surface.

The flow direction around the bubble is considered to be dependent on the temperature gradient around the bubble because the flow generation has been primarily attributed to the Marangoni force induced by localized and intense thermoplasmonic heating^33^. Because the bubble generated on the single laser spot is continuously heated from the side touched to the GNF, it is exposed to a steep temperature gradient in the direction normal to the GNF surface. Consequently, the bubble induces rapid flow in the direction normal to the substrate surface. To generate flows in the direction parallel to the substrate surface, we need to apply a temperature gradient in that direction on the bubble surface. If the bubble is fixed on the GNF, it is realized by displacing the laser spot from the bubble center^37–39^. However, because the water vapor bubble is only stable on the laser spot, the bubble follows its motion on the GNF surface and is always exposed to a temperature gradient normal to the substrate surface. Using a single laser spot, it is difficult to break the axisymmetric temperature gradient around the substrate surface normal.

In our study, to control the temperature gradient around the bubble, we focused our attention on the laser irradiation at multiple spots using a spatial light modulator (SLM)^29,40,41^. The SLM allows us to modify the wavefront of the laser beam and to design the final laser spot distributions at the focal plane. By adding a sub laser spot next to the water vapor bubble created on the primary laser spot, the bubble can be exposed to additional temperature gradients in the direction parallel to the substrate wall. We expect that the sub laser spot will facilitate in generating a rapid flow in the direction parallel to the substrate surface. In this study, we experimentally investigate the dependence of the flow field around a water vapor bubble on the power of the sub laser spot using the SLM. In addition, from the observed flow field around the bubble, we evaluate the force inducing the flow using a theoretical model and show its potential as the source of a point force in the direction parallel to the wall at the wall vicinity.

## Results

### Flow direction control via two spots laser irradiation

**Figure 1:**
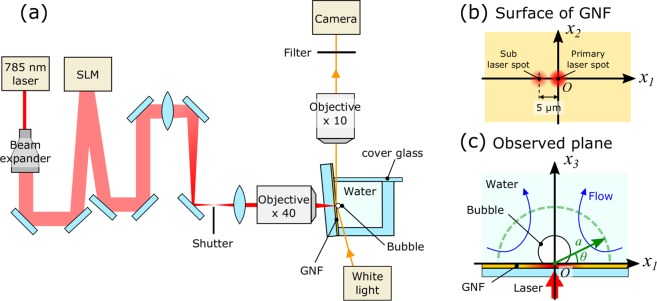
(**a**) Schematic drawings of the experimental setup. The laser irradiation at multiple spots on the GNF was realized using SLM. (**b**) Schematic drawings of the laser spot positions on the surface of GNF. (**c**) Schematic drawings of the observed region on the focal plane of the microscope. All the laser spots on the GNF are tuned to appear along the line of intersection between the focal plane of microscope and the surface of the GNF, which is indicated by axis *x*_1_.

**Figure 2:**
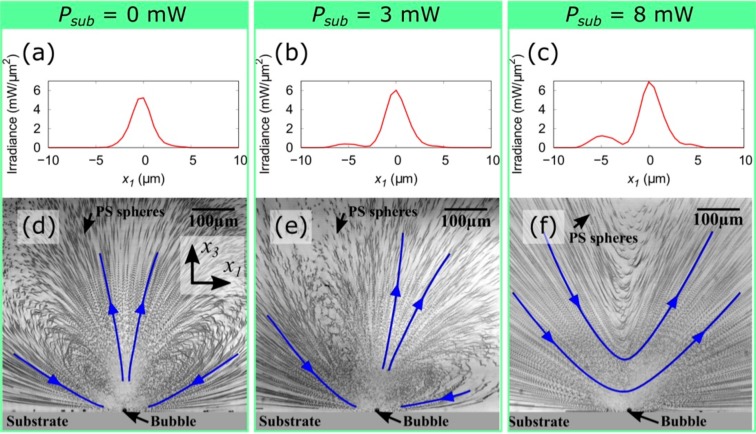
(**a**–**c**) Measured irradiance of the laser spots on *x*_1_ axis when *P*_*sub*_ = 0, 3, and 8 mW, respectively. (**d**–**f**) Observed flow around the water vapor bubble in degassed water under laser irradiation conditions of *P*_*sub*_ = 0, 3, and 8 mW, respectively. A series of 200 images captured during 1 s were merged to trace the PS spheres motion in the well-developed flow (see Supplementary Movies S1–S3).

The flow around a water vapor bubble under laser irradiation at multiple spots was observed using the experimental setup illustrated in Fig. 1a (see the Methods section for details). Briefly, a GNF was fabricated on a glass substrate using a dynamic oblique deposition technique. Subsequently, a fluidic cell was prepared on the GNF and filled with degassed water. The laser irradiation at multiple spots on the GNF was performed using an SLM^40,41^. The laser spot positions on the GNF were defined using the Cartesian coordinate system with axes *x*_1_, *x*_2_, and *x*_3_, as shown in Fig. 1b,c (see the Methods section for details). A primary laser spot for the bubble formation was irradiated at ***x*** = (0, 0, 0) on the GNF, which had a fixed power of *P*_*primary*_ = 43 ± 1 mW. Subsequently, a sub laser spot was added at ***x*** = (−5 μm, 0, 0) to provide additional temperature gradient on the bubble surface along the *x*_1_ axis. The sub laser spot position was chosen to be the same as the typical radius of the water vapor bubble^33^. The larger the distance between primary and sub laser spots, the weaker the effect of sub laser spot (see Supplementary Fig. S1). The power of the sub laser spot, *P*_*sub*_, was chosen to be 0, 3, 8, 15, and 27 mW as listed in Table 1. Figure 2a–c show the measured irradiance of the laser on the *x*_1_ axis for different laser irradiation conditions; *P*_*sub*_ = 0, 3, and 8 mW. At *x*_1_ = 0, the primary laser spots with the total laser power of 43 ± 1 mW were successfully formed in those irradiation conditions. In addition, the sub laser spots were formed at *x*_1_ = −5 μm and its power was varied to be 0, 3, and 8 mW. The typical bubble and flow generated under these laser irradiation conditions in degassed water are shown in Fig. 2d–f. The small black dots are the polystyrene (PS) spheres added to visualize the fluid motion. A series of 200 images captured during 1 s are merged to trace the motion of the PS spheres in the well-developed flow. When *P*_*sub*_ = 0, 3, and 8 mW, a water vapor bubble of 10–12 μm diameter was generated on the primary laser spot due to the highly localized heat induced by the GNF and the absence of dissolved gases in water (see Supplementary Fig. S2). The bubble demonstrated a rapid but stable flow under continuous laser irradiation as indicated by the trajectory of each PS sphere in Fig. 2d–f. The blue arrows indicate the flow direction. During the flow generation, the average temperature rise of the fluid in the cell is less than several Kelvin because the thermoplasmonic effect only induces localized heating at the vicinity of the bubble. When we irradiated only the primary laser spot on the GNF, namely *P*_*sub*_ = 0 mW (Fig. 2d), water was drawn to the bubble along the substrate surface and ejected in the direction normal to the substrate surface. In other words, the primary water stream was generated in the positive direction of the *x*_3_ axis. This result is consistent with our previous report^33^. Meanwhile, when we added a sub laser spot next to the primary laser spot, the direction of the flow around the bubble showed a significant change. When *P*_*sub*_ = 3 mW (Fig. 2e), the primary water stream generated from the bubble was tilted by ~25° to the positive direction of the *x*_1_ axis from the *x*_3_ axis. Furthermore, when *P*_*sub*_ = 8 mW (Fig. 2f), the primary water stream was tilted by ~45° to the positive direction of the *x*_1_ axis from the *x*_3_ axis. These results indicate that the flow direction around the bubble can be controlled by tuning the strength of the sub laser spot. Indeed, the further increase in *P*_*sub*_ generates a slightly larger bubble diameter with a closer position to the sub laser spot (see Supplementary Fig. S2). This can be explained by the fact that the sub laser spot starts to contribute to the bubble nucleation. Nevertheless, a stable flow similar to that shown in Fig. 2f was observed up to *P*_*sub*_ = 15 mW (see Supplementary Fig. S3). Finally, at *P*_*sub*_ = 27 mW, the bubble size became unreproducible and the flow unstable. The flow observed around the bubble is expected to be generated by the steep temperature gradient induced on the tiny water vapor bubble. However, the temperature gradient is difficult to predict because it depends on various factors, such as the bubble position relative to the laser spots, the bubble size, and the flow around the bubble. Therefore, in the next section, we will evaluate the force applied by the water vapor bubble on water along the *x*_1_ and *x*_3_ directions. The force will be assessed using the measured flow speed distribution around the bubble in the range of *P*_*sub*_ = 0–15 mW.

**Table 1:** List of laser irradiation conditions and corresponding fitting results of flow analysis.

ID	*P*_*primary*_ (mW)	*P*_*sub*_ (mW)	*F*_1_ (μN)	*F*_3_ (μN)
I	43	0	1.1 × 10^−4^ ± 2.3 × 10^−4^	1.1 × 10^−1^ ± 3.9 × 10^−3^
II	42	3	3.1 × 10^−3^ ± 2.7 × 10^−4^	4.6 × 10^−2^ ± 4.8 × 10^−3^
III	44	8	1.2 × 10^−2^ ± 2.1 × 10^−4^	6.1 × 10^−3^ ± 3.7 × 10^−3^
IV	42	15	1.1 × 10^−2^ ± 2.3 × 10^−4^	1.7 × 10^−2^ ± 3.9 × 10^−3^
V	42	27	—	—

## Discussion

To conduct a quantitative evaluation of those forces applied to water by the locally heated bubble, we introduce a simple theoretical model. In our previous study, we proved that the force induced by the water vapor bubble generated on the single laser spot was well described by a point force, namely the “stokeslet” that was placed near the surface of the GNF and whose direction was fixed normal to the surface^33^. This was because the bubble felt the temperature gradient only in the direction normal to the surface. Meanwhile, in this study, we added the component parallel to the substrate surface for the stokeslet because the sub laser spot provides a temperature gradient on the bubble in that direction. We use the Cartesian coordinate system with axes *x*_1_, *x*_2_, and *x*_3_ defined in Fig. 2b,c for the model. The surface of the GNF, i.e., the *x*_1_*x*_2_ plane, is replaced by a smooth and infinity wall with no slip. The region *x*_3_ > 0 is filled with water, which is assumed to satisfy the Stokes flow equations. Instead of the bubble, we placed the stokeslet, ***F*** = (*F*_1_, *F*_2_, *F*_3_) at ***h*** = (0, 0, *h*), near the surface of the GNF. The values of *F*_1_, *F*_2_, and *F*_3_ represent the *x*_1_, *x*_2_, and *x*_3_ components of the stokeslet strength, respectively. The analytical solution of the flow vector distribution given by a stokeslet near a non-slip wall was reported by Blake and Chwang^42^. The flow speed, ***u*** = (*u*_1_, *u*_2_, *u*_3_) at ***x*** = (*x*_1_, *x*_2_, *x*_3_) can be written as,

1 $$\begin{array}{ccc}{u}_{i} & = & \frac{{F}_{j}}{8\pi \mu }\,[(\frac{{\delta }_{ij}}{r}+\frac{{r}_{i}{r}_{j}}{{r}^{3}})-(\frac{{\delta }_{ij}}{R}+\frac{{R}_{i}{R}_{j}}{{R}^{3}})\\  &  & +\,2h\,({\delta }_{j\alpha }{\delta }_{\alpha k}-{\delta }_{j3}{\delta }_{3k})\frac{{\rm{\partial }}}{{\rm{\partial }}{R}_{k}}\,\{\frac{h{R}_{i}}{{R}^{3}}-(\frac{{\delta }_{i3}}{R}+\frac{{R}_{i}{R}_{3}}{{R}^{3}})\}],\end{array}$$

where *i* = 1, 2, and 3, *μ* denotes the viscosity of the water, *α* = 1, 2, ***r*** = (*r*_1_, *r*_2_, *r*_3_) = ***x*** − ***h***, ***R*** = (*R*_1_, *R*_2_, *R*_3_) = ***x*** + ***h***, *r* = |***r***|, and *R* = |***R***|.  *δ*_*ij*_ is the Kronecker delta; its value is 0 for *i* ≠ *j* and 1 for *i* = *j*. In equation (1), the first term in the square bracket represents the stokeslet, the second term represents a mirror image of the stokeslet against the wall, and the third includes higher-order singularities at the mirror position.

**Figure 3:**
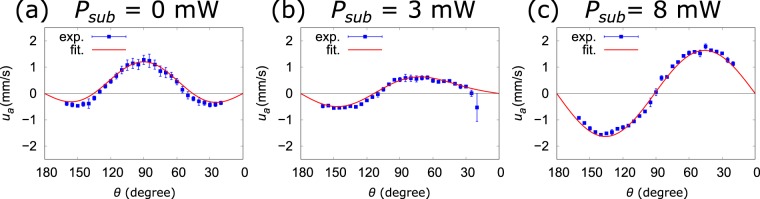
The blue squares show the measured set of *u*_*a*_(*θ*) at *a* = 250 μm. The red lines are the fitting results of equation 2 to the measured *u*_*a*_(*θ*). (**a**–**c**) Correspond to the results of the irradiation conditions of *P*_*sub*_ = 0, 3, and 8 mW, respectively.

**Figure 4:**
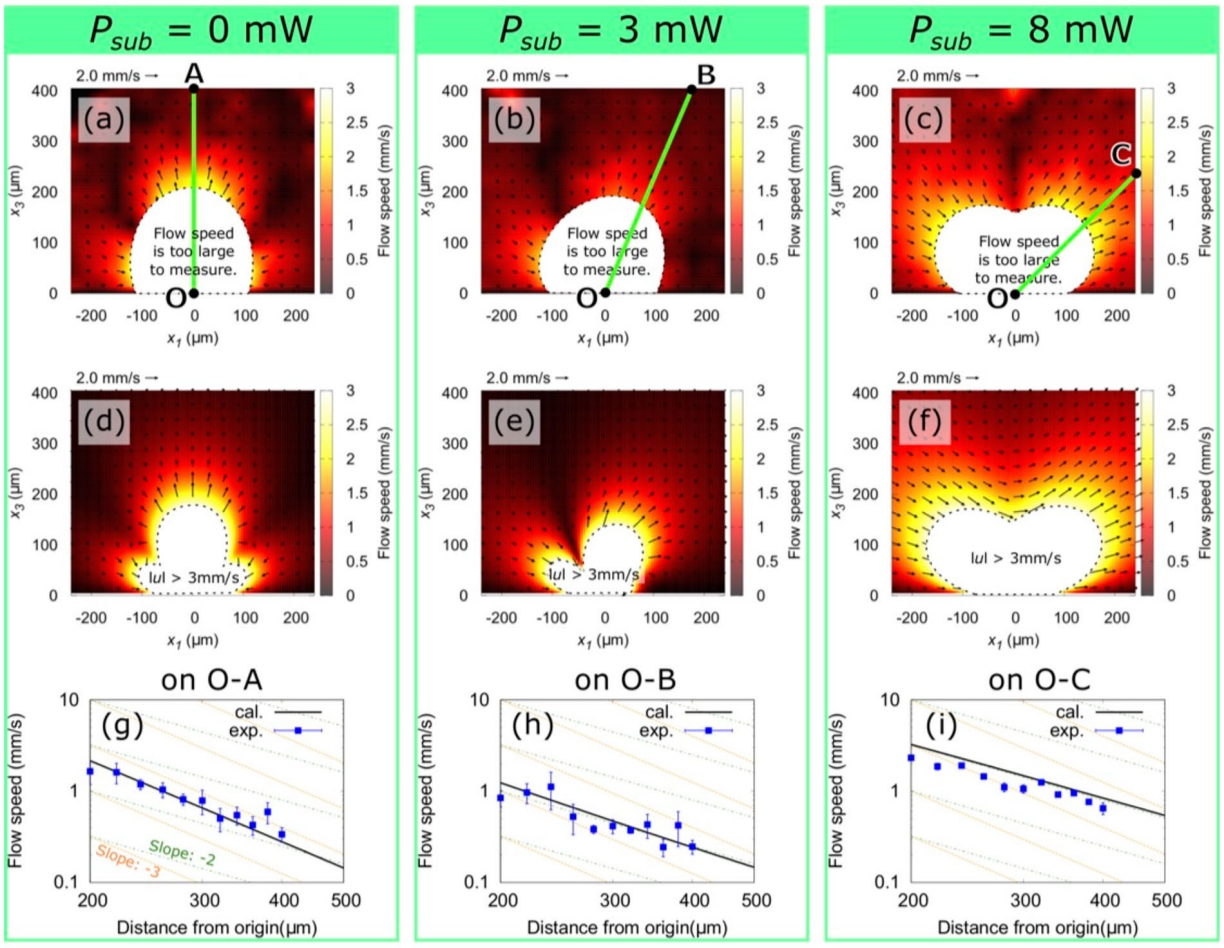
(**a**–**c**) Measured flow vectors (black arrows) and speed distribution (color map) in the region corresponding to Fig. 2d–f, respectively. (**d**–**f**) Calculated flow vectors and speed distribution around a stokeslet, whose strength is listed in Table 1. The measurements and calculations show excellent agreement. (**g**–**i**) Comparison of the radial component of the experimental data along the lines O–A/B/C (*θ* = 90°, 67°, 45°, respectively) depicted in (**a**–**c**) with the calculated results.

By fitting equation (1) to the experimentally measured flow speed distribution, the value of ***F*** can be estimated. However, equation (1) is too complicated to fit. Therefore, we simplify the equations in the far field^42^ and employ the polar coordinates system. Let us consider the flow field within the observed region, namely the *x*_1_*x*_3_ plane. The polar coordinates system is defined in Fig. 1c by the green arrow, where *x*_1_ = *a* cos *θ* and *x*_3_ = *a* sin *θ*. In the far field, the equation (1) can be simplified to be

2 $${u}_{a}=\frac{{F}_{1}}{8\pi \mu }\frac{12h\,\sin \,\theta \,\cos \,\theta }{{a}^{2}}-\frac{{F}_{3}}{8\pi \mu }\frac{12{h}^{2}\,\sin \,\theta \,\cos \,2\theta }{{a}^{3}},$$

3 $${u}_{\theta }=-\,\frac{{F}_{3}}{8\pi \mu }\frac{6{h}^{2}\,{\sin }^{2}\,\theta \,\cos \,\theta }{{a}^{3}},$$

where *u*_*a*_ and *u*_*θ*_ represent the radial and tangential components of the flow speed within the *x*_1_*x*_3_ plane, respectively. We assumed that *F*_2_ = 0 because an effective temperature gradient does not exist on the bubble in the *x*_2_ axis direction. From equations 2 and 3, it is obvious that the far-field flow induced by force components *F*_1_ and *F*_3_ fall off as *O*(*a*^−2^) and *O*(*a*^−3^), respectively. If we set the value of *μ*, *h*, and *a* in equation 2, we can fit the equation via *F*_1_ and *F*_3_ to the measured set of *u*_*a*_(*θ*). Because the water around the bubble should be well stirred and superheated^25^, a constant value of 2.8 × 10^−4^ Pa · s (at 100 °C)^43^ is chosen for *μ* to avoid the over estimation of *F*_1_ and *F*_3_. The value of *h* should be in the range of 0 < *h* ≤ *d*, where *d* is the diameter of the bubble. In our study, we choose *h* = *d* = 10 μm to avoid the over estimation of *F*_1_ and *F*_3_. Because we are considering the far field, we have chosen the reference *u*_*a*_(*θ*) at *a* = 250 μm, which is the outermost region of the observation area. The blue squares in Fig. 3a–c show the measured *u*_*a*_(*θ*) at *a* = 250 μm for *P*_*sub*_ = 0, 3, and 8 mW, respectively. The flow speed was extracted from the time sequence images of the flow using particle tracking velocimetry (LabVIEW, National Instruments). The red lines are the fitting results of equation 2 to the experimental data via *F*_1_ and *F*_3_, which shows good agreement with the experimental data. The obtained values of *F*_1_ and *F*_3_ are shown in Table 1. By substituting those values into equation 1, the flow velocity distribution within the entire observation area is calculated. Figure 4a–f show the measured and calculated flow vector and speed distribution of the entire observed region, respectively. The flow speed is represented by color, and the flow direction is indicated by black arrows. Although the values of *F*_1_ and *F*_3_ are obtained under the far-field approximation, the measurements and calculations show good agreement. A closer comparison between the calculation and experimental results was performed by extracting the results on the radial lines O-A/B/C indicated in Fig. 4a–c. These lines are chosen to avoid the critical region, where the flow velocity changes significantly. The measured flow speed indicated by blue squares agrees well with the calculation indicated by the black lines (Fig. 4g–i). These results suggest that the model describes well the observed flow around the water vapor bubble.

**Figure 5:**
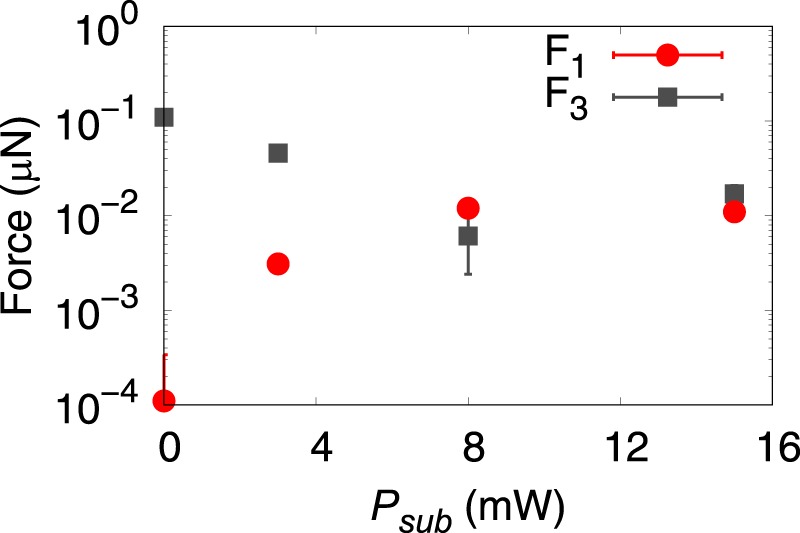
The fitting results of *F*_1_ (red solid circle) and *F*_3_ (gray solid square) as a function of *P*_*sub*_. These values are listed in Table 1.

Because the model described the experimental results excellently, the calculation results are more detailed. The values of *F*_1_ and *F*_3_ listed in Table 1 were plotted in Fig. 5 as a function of *P*_*sub*_. When *P*_*sub*_ = 0 mW, the fitted value of *F*_3_ was three orders of magnitude larger than *F*_1_. In addition, the flow field in the region 200 < *a* < 500 μm is dominated by the component of *F*_3_ because the decay rate of the flow speed is *O*(*a*^−3^), as shown in Fig. 4g. This result is reasonable given that the primary laser spot generates the water vapor bubble and provides a steep temperature gradient on the bubble along the *x*_3_ axis due to the highly localized thermoplasmonic effect of the GNF. This temperature gradient induces the Marangoni force on the bubble surface along the positive direction of the *x*_3_ axis^33^, namely *F*_3_. When *P*_*sub*_ is in the range of 0–8 mW, *F*_1_ increases proportionally to *P*_*sub*_, while *F*_3_ is inversely proportional to *P*_*sub*_ (Fig. 5). The increase of *F*_1_ may be attributed to an increase of the effective temperature gradient along the *x*_1_ axis. In that range, the bubble is generated on the primary laser spot (see Supplementary Fig. S2) because *P*_*sub*_ is smaller compared to the bubble nucleation threshold of ~10 mW^33^. Although the sub laser spot contributes insignificantly to the bubble generation, it yields an effective temperature gradient on the bubble along the *x*_1_ axis. This temperature gradient induces a force in the positive direction of the *x*_1_ axis, which becomes prominent as the power of the sub laser spot increases. Then, both *F*_1_ and *F*_3_ are saturated at the order of 10^−2^ μN as they show insignificant change between *P*_*sub*_ = 8 mW and 15 mW. As *P*_*sub*_ exceeds the bubble nucleation threshold, the bubble starts to deviate from the primary laser spot towards the sub laser spot position (see Supplementary Fig. S2). This bubble position shift may cancel the effective temperature gradient along the *x*_1_ axis to limit increasing *F*_1_. Therefore, further investigation is required for better understanding. At this stage, the decline rate of the flow speed around the bubble is *O*(*a*^−2^), which indicates that *F*_1_ dominates the flow field in the considered region (Fig. 4i). Although the temperature gradient around the bubble is significantly affected by the resulting flow field and is difficult to estimate, the model proved that we could successfully induce a stokeslet in the direction parallel to the substrate surface.

**Figure 6:**
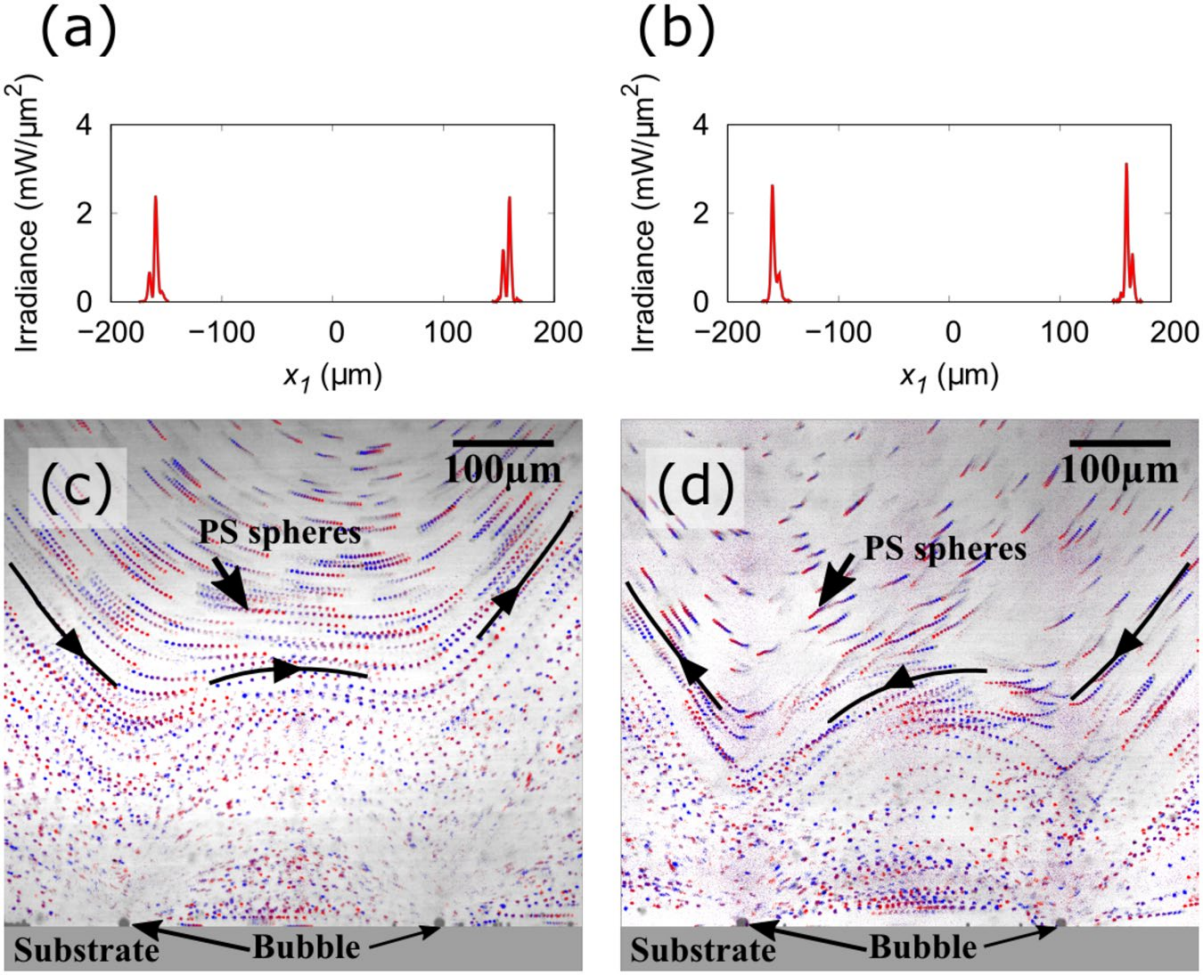
(**a** and **b**) Measured irradiance of the laser spots on *x*_1_ axis. Whereas the primary laser spots remain fixed to *x*_1_ = ±160 μm, the sub laser spots positions were switched from (**a**) *x*_1_ = −165 μm and 155 μm to (**b**) *x*_1_ = −155 μm and 165 μm. (**c** and **d**) Observed flow around the water vapor bubble in degassed water under laser irradiation conditions of (**a** and **b**), respectively. A series of 10 images captured during 0.05 s were merged to trace the PS spheres motion in the well-developed flow. The string color gradation in the PS sphere images represents the flow direction (see Supplementary Fig. S4). It changes depending on the sub laser spot positions (see Supplementary Movies S4 and S5).

To demonstrate the practicability of our technique, we performed flow generation using two bubbles. We irradiated two pairs of laser spots, where each pair includes the primary and sub laser spots. Figure 6a,b show the measured irradiance of the laser on the *x*_1_ axis. Whereas the primary laser spots remain fixed to *x*_1_ = ±160 μm, the sub laser spots positions were switched from *x*_1_ = −165 μm and 155 μm (Fig. 6a) to *x*_1_ = −155 μm and 165 μm (Fig. 6b). Figure 6c,d show the typical flow generated by the laser irradiation conditions shown in Fig. 6a,b, respectively. The colored small dots and strings are the PS spheres trajectories. A series of 10 images were captured over 0.05 s and merged to trace the PS spheres motion in the liquid flow. The string color gradation in the PS sphere images represents the time sequence, where each sphere moves from the blue to red position in 0.05 s (see Supplementary Fig. S4). On each primary laser spot, a water vapor bubble was generated and generated rapid flow. Because the bubbles shown in Fig. 6c generate forces in the positive direction of the *x*_1_ axis, they generate rapid flows parallel to the substrate surface in that direction. Only by switching the position of the sub laser spot from left to right of the primary laser spots, the flow direction changed in the opposite direction (Fig. 6d). The flow direction is switchable repeatedly between the directions shown in Fig. 6c and that of Fig. 6d. These flow patterns are significantly different from that observed under similar laser irradiation condition with *P*_*sub*_ = 0 mW, where the flows around the bubbles interfere and depress each other (see Supplementary Fig. S5). In addition, we can obtain the flow speed of at least 7 mm/s at a distance of 100 μm from the substrate surface by manually tracking the motion of the PS spheres shown in Fig. 6c, which is the maximum flow speed measurable using our camera. In the vicinity of the bubbles, where the distance from the substrate surface is even smaller than 100 μm, the flow speed is expected to be several orders of magnitude larger than the measured flow speed. Through the asymmetric heating method proposed in this study, the flow around the water vapor bubble is convenient for microfluidic pumping, whose flow direction is tunable flexibly by designing a laser spot pattern. Furthermore, our method yields a peculiar flow field, in which a strong shear flow is generated at the wall vicinity, compared to the general pressure-driven flow field in channels, where the flow speed is reduced at the wall vicinity because of the fluid viscosity. Therefore, the method is expected to be useful not only for pumping but also for the enhancement of heat exchange between a solid wall and fluid, chemical reactions on the wall, etc. Finally, let us remember that the heat source for the bubble and the corresponding flow generation is not limited to photothermal heating used in this study. It provides spatiotemporally flexible heating, which allows carrying out systematic experiments and optimizing the heating pattern to generate a desired flow. After optimization, the photothermal heating can be replaced by other heating methods, such as thin film metal heaters that are reliable, cost-effective, and electrically controllable for rapid microfluidic manipulation. These features may allow us to integrate our technique in programmable digital microfluidic devices^44,45^ and broaden its practical applicability. Our future work includes the heating pattern optimization for flow generation, the evaluation of pumping efficiency, and the enhancement of chemical reactions using rapid flow in order to apply our technique in microfluidic systems, such as single cell analyzers^46^ and drug discovery platforms^47^.

## Conclusion

In summary, we investigated the rapid flow generated around a water vapor microbubble using the thermoplasmonic effect of the GNF under laser irradiation with multiple spots. A single primary laser spot focused on the GNF generated a water vapor bubble with a diameter of ~10 μm in degassed water because of the highly localized heat generation of the GNF. Simultaneously, the laser spot applied a steep temperature gradient in the direction normal to the substrate surface on the bubble and induced a rapid flow in that direction. By adding a sub laser spot next to the primary laser spot, a temperature gradient in the direction parallel to the substrate surface was applied on the bubble. Consequently, the flow direction around the bubble tilted from the direction normal to the substrate surface, and a rapid flow in the direction parallel to the substrate surface was generated. The observed flow around the water vapor bubble was well described using a stokeslet, which contained components normal and parallel to the substrate surface and was set to 10 μm above the GNF. The model suggested that the parallel component can dominate the flow by optimizing the sub laser spot power. Overall, this technique allowed us to apply a force on the microfluid at the vicinity of the wall, where the flow speed was generally suppressed by viscosity, in the direction parallel to the wall surface. This technique is expected to be useful for microfluidic pumping, enhancement of heat exchanges, and enhancement of chemical reactions at the vicinity of the wall.

## Methods

### Preparation of gold nanoisland films

The GNF was prepared using a glancing angle deposition technique. Gold was deposited on a glass substrate until an average thickness of 10 nm, during which the substrate was held at an oblique angle of 73.4° and rotated continuously and rapidly^33^. The prepared thin film was subsequently placed in a UV ozone cleaner (UV253S, Filgen) for 30 min to improve the surface wettability. The optical absorption of the GNF at the wavelength of 785 nm was found to be 0.31 from the optical reflectance and transmittance measurements using a single-beam spectrophotometer and an integration sphere (ISP-REF, Ocean Optics).

### Preparation of degassed water

We prepared degassed water, in which PS spheres were dispersed to visualize the flow. The water suspension of the PS spheres with a diameter of 2 μm (R0200, Thermo Scientific) was diluted with ultrapure water (18.2 MΩ cm from Millipore-Direct Q UV3, Merck) to a ratio of 1:200 (particle number density: ~1 × 10^7^ cm^−3^). Subsequently, the diluted suspension was sonicated under a water aspirator vacuum (~3 kPa at 25 °C) for 20 min. The dissolved oxygen concentration in the degassed water is 0.9 ± 0.1 mg/L (measured by DO-5509, FUSO). The degassed water was carefully transferred into a fluidic cell of 10-mm cube created on the GNF and sealed with a cover glass. The following microfluidic observation was performed within 20 min after the cell preparation to avoid diffusing air into the water.

### Observation of bubble formation and Marangoni flow

The prepared fluidic cell was placed in our observation system, which includes an upright microscope (M-scope, Synos) for observation and a laser irradiation system for thermoplasmonic heating (Fig. 1a). The upright microscope used for the observation of the fluidic phenomena in the cell was equipped with an objective lens (10×, NA = 0.26). The observed region was on the focal plane of the microscope, which was normal to the surface of the GNF and had a thickness of ~20 μm^33^. The surface of the GNF was tilted by 5° from the optical axis of the microscope to confirm the laser spot on the film. The bubble formation and fluid motion visualized by the motion of PS spheres were recorded using a CMOS camera (HXC20, Baumer), whose exposure time and frame rate were set to 0.5 ms and 200 fps, respectively. A short-pass filter was placed in front of the camera to eliminate the 785-nm laser source that was used for thermoplasmonic heating.

The laser irradiation at multiple spots on the GNF was performed using an SLM^40,41^. First, a laser beam (CW, wavelength 785 nm) was enlarged using a beam expander and reflected by the SLM (LCOS-SLM, X10468-02, Hamamatsu Photonics) to control the wavefront of the beam. The phase hologram used for the wavefront control was calculated by the iterative Fourier transform method. The modified laser beam propagated through a telescope (the magnification was approximately 0.3) during which unnecessary light was eliminated by a shutter placed around the beam waist in the telescope^41^. The beam diameters immediately before the objective lens were approximately 2.1 mm. Finally, the beam was introduced to the objective lens (40×, NA = 0.60) to form the desired laser spots on the GNF.

To define the laser spot positions on the GNF, we applied the Cartesian coordinates system with axes *x*_1_, *x*_2_, and *x*_3_ to the fluidic cell. Following Fig. 1b,c, axes *x*_1_ and *x*_2_ are along the surface of the GNF, and the axis *x*_3_ is normal to the surface. The region *x*_3_ > 0 is filled with water. In addition, we choose the axes *x*_1_ and *x*_3_ to be within the observed region of the microscope. A primary laser spot for the bubble formation was irradiated at ***x*** = (0, 0, 0) on the GNF, which had a fixed power of *P*_*primary*_ = 43 ± 1 mW. Subsequently, a sub laser spot was added at ***x*** = (−5 μm, 0, 0) to provide an additional temperature gradient on the bubble surface along the *x*_1_ axis. The position of the sub laser spot was chosen to be the same as the typical radius of the water vapor bubble^33^. The power of the sub laser spot, *P*_*sub*_, was varied from 0 to 27 mW. Each laser spot exhibits a Gaussian distribution and its full width at half maximum of those laser spots was 2–3 μm.

## Supplementary information

Supplementary Information

Supplementary Information

Supplementary Information

Movie S1

Movie S2

Movie S3

Movie S4

Movie S5

Supplementary Information
