# Supplementary material for: Direction control of quasi-stokeslet induced by thermoplasmonic heating of a water vapor microbubble
Source: Sci Rep. 2019 Mar 18;9:4770. doi: 10.1038/s41598-019-41255-5 (PMC6423281; doi:10.1038/s41598-019-41255-5)
Supplement: Supplementary file 9 — Supplementary Information [file 41598_2019_41255_MOESM9_ESM.pdf]

Direction control of quasi-stokeslet induced by thermoplasmonic heating of a water vapor microbubble

Kyoko Namura,*,† Souki Imafuku,† Samir Kumar,† Kaoru Nakajima,† Masaaki Sakakura,‡ and Motofumi Suzuki†

†*Department of Micro Engineering, Kyoto University, Kyoto Daigaku-Katsura, Nishikyo-ku, Kyoto 615-8540, Japan*

‡*University of Southampton, Optoelectronics Research Centre, Southampton, SO17 1BJ, United Kingdom*

E-mail: namura@me.kyoto-u.ac.jp

Phone: +81 75 383 3697. Fax: +81 75 383 3697

Supplementary information

1. Movies

Movies S1–S5 show the flow around the water vapor bubbles under different laser irradiation conditions. The observed region is approximately 570 µm × 500 µm and nearly normal to the gold nanoisland film surface. The small black dots are the polystyrene spheres with a diameter of 2 µm, which have been added to aid the visualization of fluid motion.

Movies S1–S3 correspond to Fig. 2d–f. A set of primary and sub laser spots is irradiated on the gold nanoisland film. The flow direction around a bubble changes according to the sub laser spot power.

Movies S4 and S5 correspond to Fig. 6c, d. We irradiated two pairs of laser spots; each pair includes the primary and sub laser spots. Only by changing the position of the sub laser spot from the left to the right of the primary laser spots, the flow direction changes to the opposite direction.

2. Figures

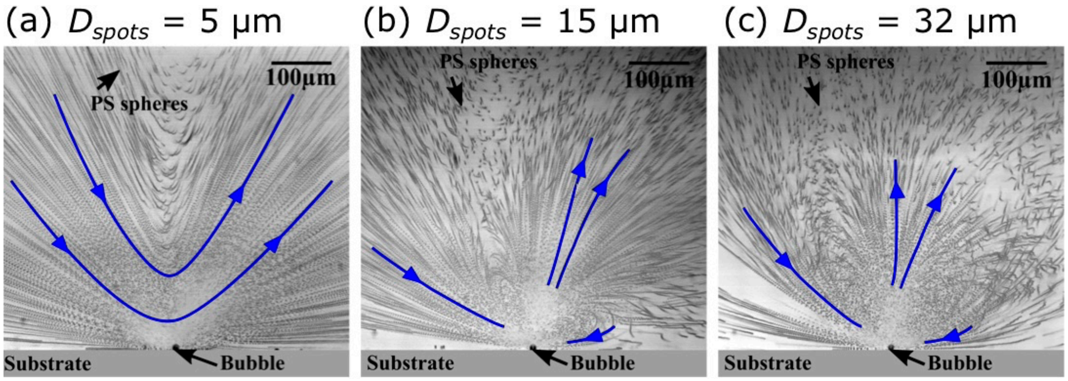

Figure S1: Dependence of the flow around the water vapor bubble on the distance between primary and sub laser spots, D_{spots}. A series of 200 images captured in 1 s are merged to trace the PS spheres' motion in the well-developed flow. Power of the primary laser spot, $P_{primary}$, and the sub laser spot, P_{sub}, are fixed to 43±1 mW and 9±2 mW, respectively. As D_{spots} increases from 5 μm to 32 μm, the flow pattern becomes increasingly similar to that observed at $P_{sub} = 0$ mW (Fig. 2d). This can be explained by the disappearance of the sub laser spot effect on the flow generation as the spot leaves the bubble surface, which has a typical radius of 5 μm.

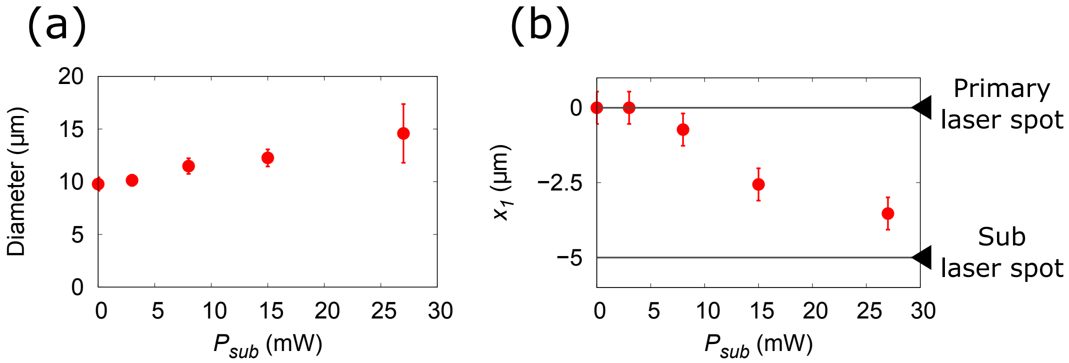

Figure S2: (a) Bubble diameter as a function of P_{sub}. At P_{sub} = 0 mW–15 mW, the bubble diameter increased slightly as P_{sub} increased. The bubble size is unstable at P_{sub} = 27 mW. (b) Bubble nucleation position as a function of P_{sub}. The nucleation position becomes closer to the sub laser spot as the sub laser power increases.

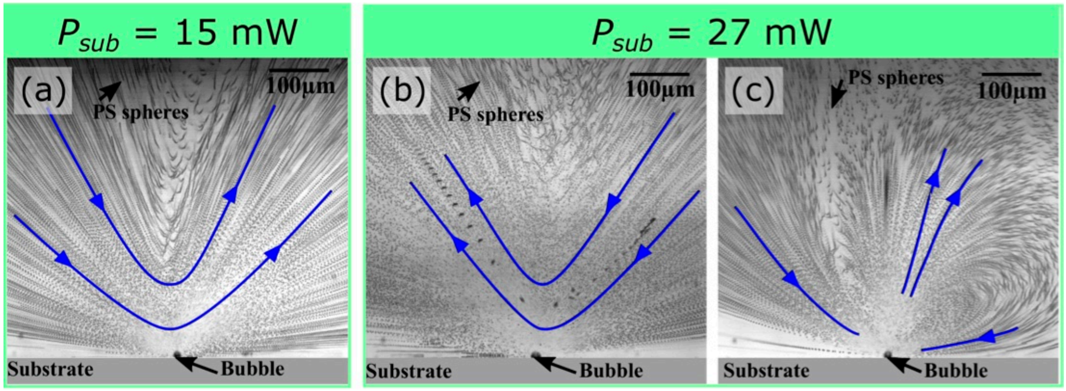

Figure S3: Observed flow around the water vapor bubble in degassed water at (a) P_{sub} = 15 mW and (b,c) P_{sub} = 27 mW. A series of 200 images captured in 1 s are merged to trace the PS spheres' motion in the well-developed flow. At P_{sub} = 15 mW, the flow around the bubble is stable and similar to that observed at P_{sub} = 8 mW (Fig. 2f). Flow pattern around the bubble is not reproducible at P_{sub} = 27 mW. This is because both primary and sub laser spots significantly contribute to the bubble nucleation at high P_{sub}, and the bubble size is not reproducible (Fig. S2a) .

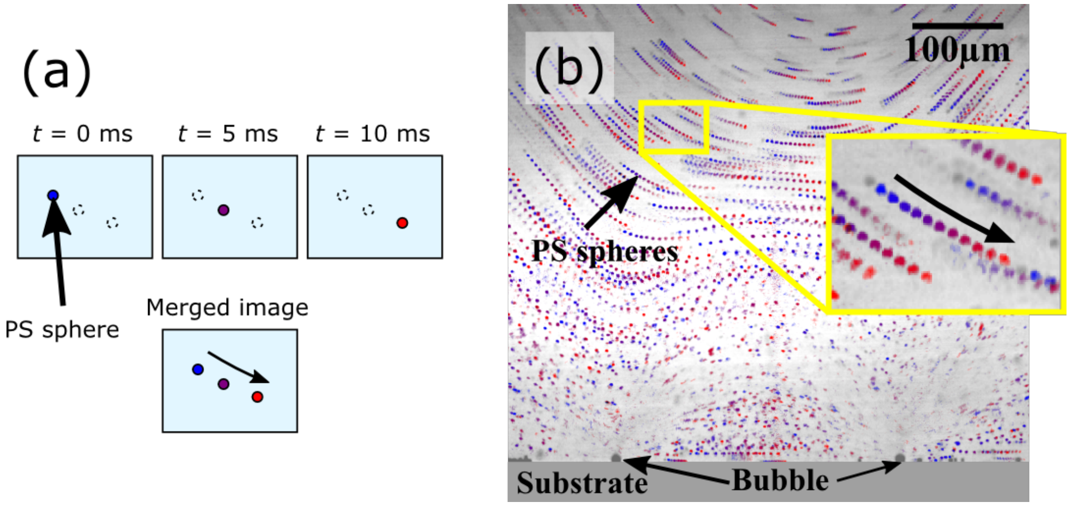

Figure S4: (a) Schematic of the method to generate the colored trajectory of the PS sphere. First, a series of images taken every 5 ms were colored from blue to red. Then, they were merged to trace the PS spheres' motion in the liquid flow. The string color gradation in the PS sphere images represents the time sequence, where it moves from the blue to red position. (b) Typical image generated by the above method. Series of ten images captured over 0.05 s are merged to trace the PS spheres' motion. The enlarged image shows one of the colored PS sphere trajectories, which indicates that the sphere has moved along the black arrow in 0.05 s.

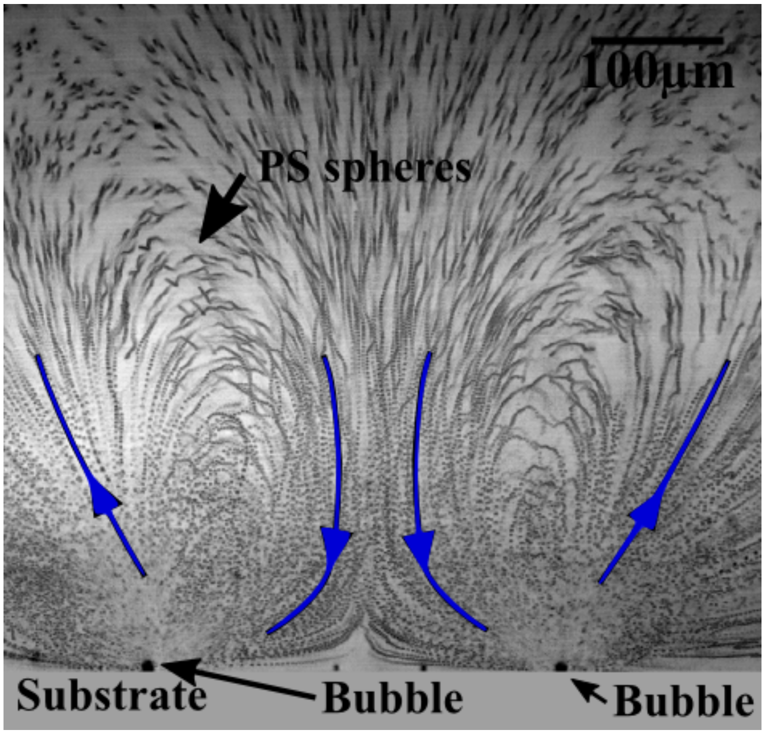

Figure S5: Observed flow generated by two water vapor bubbles. Each of them was generated on a single laser spot, and the sub laser spots were not irradiated. The positions of the laser spots were fixed to $\boldsymbol{x}$ = (±160 μm, 0, 0), whose powers 32 mW and 38 mW. A series of 200 images captured in 1 s are merged to trace the PS spheres' motion in the well-developed flow. Flows around the bubbles interfere and depress each other. The flow pattern significantly differs from that shown in Fig. 6c,d, where rapid pumping flow was observed along the substrate surface. These results suggest that the asymmetric heating proposed in this study (Fig. 6c,d) is convenient for microfluidic pumping compared to the symmetric heating proposed in our previous study[33].
